# The Sanatorium Village

**Published:** 1911-01-14

**Authors:** 


### The Sanatorium Village.

Charles Dickens said that when he landed at Boulogne the letters composing the familiar motto, "Liberty, Equality, and Fraternity," struck him as being a little too tall for their width. Somehow one is reminded of this curiously minute criticism by a neat little essay on certain sanatoriums written by a French architect, M. Richter. Dealing specially with construction, he begins with the Royal Victoria Hospital for Consumption at Edinburgh, and passes on successively to the popular sanatorium at Leysin, to that at Bligny, and finally to a rather noteworthy institution at Montigny-en-Ostrevent, of which it is surprising, seeing how much the subject is written on, that more has not been heard previously. Here, besides fifty-two ordinary beds, half for men and half for women, are provided twenty-four "villas de cure" (specially built) which accommodate families having one parent tuberculous, the invalid being, of course, provided with a separate room. But the cost, even divided over the seventy-six patients, runs up to more than 14,000 francs per bed. Nevertheless, what has suggested itself to M. Richter is whether something more extensive still cannot be done towards transplanting consumptives and their families to rural surroundings: the plan favoured being nothing less than a community complete—what he styles, with French passion for general terms, a "village-anti-bacillaire" or a "sanatorium village." He quotes a list of particulars regarding the consumptives discharged in 1908 from the institution at Bligny. The 165 cases were recruited from a good many different occupations, and moreover sixty of them had to do in some way with the building trade. Very well then, says M. Richter, these sixty can build our model open-air village, beginning with a permanent structure destined to bring under one roof the offices of administration, the kitchens and dining-halls, and the servants' quarters. The patients shall be lodged in *maisonnettes*, lightly put together and run up as required, at the rate, say, of two or three a year. Laundries, workshops, farm-buildings, doctor's and nurses' houses, a school, and many other things can follow, until ultimately the colony is complete from a church down to a rabbit-hutch. It is to surpass even the Belgian Gheel; for while there the special class of lunatics is billeted upon the inhabitants of a countryside, here the special class of consumptives is to constitute the whole population.

And here, obviously, is where the plan seems top-heavy. We have nowhere got labour colonies for the tuberculous properly established yet: when that is accomplished it will be time enough to see about further developments. Any that grow naturally out of labour colonies, just as labour colonies do from popular sanatoriums, may be welcomed; but in the present low state of anti-tuberculosis funds it would hardly be justifiable to embark on the always highly risky project of creating from nothing a small separate community. In the Countess of Aberdeen's amiable preface this point is overlooked.

